# In sync: gamma oscillations and emotional memory

**DOI:** 10.3389/fnbeh.2013.00170

**Published:** 2013-11-21

**Authors:** Drew B. Headley, Denis Paré

**Affiliations:** Center for Molecular and Behavioral Neuroscience, Rutgers, The State University of New JerseyNewark, NJ, USA

**Keywords:** gamma oscillations, emotional memory, associative memory, arousal, amygdala

## Abstract

Emotional experiences leave vivid memories that can last a lifetime. The emotional facilitation of memory has been attributed to the engagement of diffusely projecting neuromodulatory systems that enhance the consolidation of synaptic plasticity in regions activated by the experience. This process requires the propagation of signals between brain regions, and for those signals to induce long-lasting synaptic plasticity. Both of these demands are met by gamma oscillations, which reflect synchronous population activity on a fast timescale (35–120 Hz). Regions known to participate in the formation of emotional memories, such as the basolateral amygdala, also promote gamma-band activation throughout cortical and subcortical circuits. Recent studies have demonstrated that gamma oscillations are enhanced during emotional situations, coherent between regions engaged by salient stimuli, and predict subsequent memory for cues associated with aversive stimuli. Furthermore, neutral stimuli that come to predict emotional events develop enhanced gamma oscillations, reflecting altered processing in the brain, which may underpin how past emotional experiences color future learning and memory.

## Introduction

Emotions are layered upon the stream of sensations that we experience throughout our lives. Beyond being merely another event in an experience, they shape the quality, vividness, and persistence of what is remembered. The mechanisms underlying this likely encompasses processes during encoding of the experience, its subsequent consolidation, and eventually retrieval.

In principle, the neural processes that support emotional memory should exhibit similar properties to emotional memory itself. Such an isomorphism underpins virtually every attempt to construct neural explanations for psychological processes (Boring, 1936). This review will argue that gamma oscillations contribute to the persistence and vividness of emotional memories. Suggestively, and independent of any observed linkage with emotion, gamma oscillations allow for efficient transmission of information between neural ensembles, and in doing so may produce lasting changes in their connectivity. More directly, gamma oscillations are observed in numerous studies of emotional memory and processing. As reviewed below, they are generated during circumstances that incite emotional arousal and are enhanced by stimuli that predict affective events. Furthermore, much evidence indicates that gamma oscillations predict subsequent memory for emotional experiences, and the accompanying neuroplasticity. This is not to say that gamma oscillations in particular support emotional memory, but that understanding why emotional events have such a profound impact on memory requires an appreciation of how they engage gamma oscillations.

## Background

### Emotional memories: what are they?

Before addressing the neural basis of emotional memories, we must outline their properties and how they are studied. Readers familiar with these concepts can skip to the subsequent sections. Emotional memories are typified by their endurance, vividness, and ease of recall (Rapaport, 1971; Christianson and Loftus, 1987; Bradley et al., 1992; Cahill and McGaugh, 1995; Kensinger and Schacter, 2008). Operationally defined, emotional memories are long-lasting changes in behavior arising from affective experiences. Packed into this definition are two essential concepts. The first is a definition of memory itself, an experience-dependent change in behavior. In the behaviorist tradition, which is the *de facto* psychological paradigm in neuroscience, memory, like all psychological processes, is inferred by observing the actions of an organism. The other defining characteristic is the presence of affective events. What constitutes affective is deceptively simple. Affective stimuli often have a direct impact on an organism's homeostasis, either by providing nourishment or inflicting physical distress. But for many situations, the emotional stressor is just perceived or anticipated. For instance, emotional memory can arise from viewing a gruesome picture, or the delivery of bad news. Thus, an emotional experience does not require an overt biologically salient stimulus. And even if the stimulus is overtly affective, its salience is influenced by a subject's goals and circumstances; a food reward will not be arousing to a satiated subject.

The distinction between perceived and actual affective stimuli is most stark when comparing studies of emotional memory in humans and other animals. Affective stimuli in human memory studies are often images of violence or nudity (e.g., Bradley et al., 1992), at worst electric shocks, while in non-human animal studies they range from electric shocks to copulation (e.g., Pfaus et al., 2001). This systematic difference in the intensity of experiences used to study emotional memory complicates the search for a general cross-species framework. Nevertheless, we will assume that all subjects in the studies we review underwent emotional experiences.

Studies of emotional memory typically include three phases, and these will organize our coverage of the literature (Table 1). The first phase is *acquisition*, when subjects are exposed to the circumstances to be remembered. In most human studies, subjects are exposed to multiple stimuli, some neutral and others emotional, and they must remember which stimuli they were exposed to. For other species, a subject learns that particular neutral stimuli precede emotional ones. Pervading the description of these training paradigms is Pavlovian terminology, which will be used throughout this review (Pavlov, 1927). Neutral stimuli lacking inherent motivational properties are referred to as conditioned stimuli (CS). If multiple CSs are present, then a stimulus that predicts an affective event it is called a CS+, while one that does not is a CS−. Inherently affective stimuli are referred to as unconditioned stimuli (US).

**Table 1:** **Summary of cited literature on gamma oscillations and emotional stimuli**.

**Citation**	**Species**	**Emotional**	**Effect**
		**valence**	**on gamma**
**PASSIVE VIEWING**			
Freeman, 1960	Cat	Positive	↑
Müller et al., 1999	Human	Positive/negative	↑
Keil et al., 2001	Human	Positive/negative	↑
Oya et al., 2002	Human	Positive/negative	–/↑
Matsumoto et al., 2006	Human	Negative	↑
Balconi and Pozzoli, 2007	Human	Negative	↑
Luo et al., 2007	Human	Negative	↑
Basar et al., 2008	Human	Positive	–
Glauser and Scherer, 2008	Human	Positive/negative	↓
Oathes et al., 2008	Human	Negative	↑
Luo et al., 2009	Human	Negative	↑
Siegle et al., 2010	Human	Negative	↑
Jung et al., 2011	Human	Positive/negative	↑/↓
Sato et al., 2011	Human	Positive/negative	↑/–
Senkowski et al., 2011	Human	Positive/negative	↑
Martini et al., 2012	Human	Negative	↑
**ACQUISITION**			
Miltner et al., 1999	Human	Negative	↑
Keil et al., 2007	Human	Negative	↑
Jeschke et al., 2008	Gerbil	Negative	↑
Headley and Weinberger, 2011	Rat	Negative	↑
**CONSOLIDATION**			
Popa et al., 2010	Rat	Negative	–
**RETRIEVAL AND EXPRESSION**			
Dumenko, 1995	Dog	Positive	↑
Bauer et al., 2007	Cat	Positive	↑
Popescu et al., 2009	Cat	Positive	↑
Headley and Weinberger, 2013	Rat	Negative	↑

This list only covers the studies mentioned in this review, and thus should not be considered exhaustive.

The second phase is *consolidation*, wherein the memory for an experience strengthens with time, becoming less sensitive to interference and disruption (Müller and Pilzecker, 1900). Consolidation begins during the experience and persists for hours, days, perhaps even years (Squire and Cohen, 1979). Emotional states that occur during or shortly after an experience influence its consolidation through a cascade of physiological processes, often enhancing memory duration and strength (McGaugh, 2004; Izquierdo et al., 2006).

The final phase is *retrieval* or *expression*. Here subjects either explicitly express their recollection of an emotional stimulus, or behave in a way that anticipates it. For instance, hungry subjects approach cues that predict food. During this period, the memory and any associated changes in the brain are assessed.

### Cortical activation: an early neural correlate of arousal

Before discussing gamma oscillations and their role in emotional memory, it is worth revisiting a related phenomenon, electroencephalographic (EEG) activation, that received extensive investigation as a signature of emotional processing. Since at least the 1940s, it has been known that emotionally salient stimuli drive the nervous system differently. EEG studies were the first to identify the stimulus-driven neural correlates of arousal. Cortical EEG activation was one such response, referred to variously as “activation,” “desynchronization,” “low-voltage fast,” or “alpha-blocking,” because it replaced the slow EEG rhythms that accompany a relaxed state with a fast low amplitude chatter. What made the activation response compelling was that it increased with the arousing properties of the triggering stimuli and could be acquired by neutral stimuli that gained behavioral relevance (Magoun, 1958). The activation response could also diminish for stimuli that were repeatedly presented without overt reinforcement, initially encompassing the entire neocortex, but across trials retreating to the cortical region corresponding to a stimulus' particular modality (Rheinberger and Jasper, 1937; For review see: Morrell, 1961). Of relevance to emotional memory, pairing a tone with shock strengthened the cortical activation to the tone (Gluck and Rowland, 1959).

Soon, it was found that cortical activation and behavioral arousal reactions could be elicited by electrical stimulation of brain stem nuclei (Moruzzi and Magoun, 1949), which released acetylcholine throughout the cortex (Kanai and Szerb, 1965). Later studies revealed that this cholinergic component of cortical activation was produced by the nucleus basalis (NB), the dominant source of cholinergic innervation to the cortex (Mesulam et al., 1983). Electrical stimulation of the NB produces neocortical activation that is blocked by muscarinic cholinergic receptor antagonists (Metherate et al., 1992). The NB also receives projections from the amygdala, a structure long implicated in emotion (Kluver and Bucy, 1939). This pathway likely mediates the cortical activation evoked by amygdala stimulation (Dringenberg and Vanderwolf, 1996).

What does cortical activation have to do with gamma? Early investigators recognized that cortical activation is associated with the enhancement of high frequency oscillations (Jasper and Andrews, 1938). More modern work has thoroughly demonstrated that gamma oscillations suffuse the activated cortical state (Steriade et al., 1996). Therefore, studies of cortical activation between the thirties and sixties were likely the first systematic, albeit unintended, investigations of gamma oscillations during emotional experiences.

### Generation of gamma oscillations

By virtue of their intrinsic properties and connectivity, neuronal networks throughout the brain are prone to oscillating. Oscillations in the gamma-band, from 35 up to 120 Hz, of the local field potential (LFP) and EEG, have recently received particular attention because they co-occur with numerous psychological processes, and are attractive candidates for coordinating activity within local circuits and between distant brain regions (Fries, 2005; Wang, 2010). Their relevance to emotional behavior arises from these properties.

Stimulus-driven gamma oscillations in the cortex can be classified into two types based on a scheme devised by Galambos (1992). The first are *evoked*, defined by their consistent phase-locking with stimulus onset. This consistency leads to the evoked gamma oscillation being readily observable by averaging the neural response (EEG or LFP) across repeated stimulus presentations. They usually occur at a relatively short-latency from stimulus onset, with a laminar profile similar to potentials evoked by thalamic stimulation (Sukov and Barth, 1998). The other form of stimulus–driven gamma is *induced*, which is not time-locked with stimulus onset and typically emerges a few hundred milliseconds after evoked gamma has subsided. Induced gamma oscillations can be distributed across all cortical layers without reversing polarity (Steriade and Amzica, 1996), and their longer latency suggests a top-down origin or modulatory influence (e.g., Parikh et al., 2007).

Similar circuits produce gamma oscillations in the neocortex (Whittington et al., 2011), hippocampus (Csicsvari et al., 2003), and amygdala (Randall et al., 2011). In each case, gamma oscillations arise from the interaction between reciprocally connected groups of excitatory principal neurons and inhibitory interneurons. Given the similarities, we will focus on the neocortical circuit to describe the emergence of gamma.

Principal excitatory neurons in the neocortex are sparsely interconnected, with a connection probability of around 10% for pairs that are within 150 μm of each other (Brown and Hestrin, 2009; Oswald et al., 2009; Levy and Reyes, 2012). These connections are highly specific, with reciprocally connected pairs occurring in excess of chance. Moreover, the average connection strength in a neocortical ensemble increases with ensemble size (Perin et al., 2011). Taken together, these findings suggest that subsets of principal neurons form highly specific cellular ensembles that may be fully activated by exciting only a few of their members.

In contrast, principal neurons form extensive connections with inhibitory interneurons, peaking at 50% connection probability with their nearest neighbors (Levy and Reyes, 2012). While there is a profusion of interneuron types in the neocortex (For review see: Markram et al., 2004), we will focus on the parvalbumin positive subtype, because of its established role in neocortical gamma oscillations (Sohal et al., 2009; Buzsaki and Wang, 2012). These interneurons are highly interconnected, both with chemical and electrical synapses (gap junctions), promoting synchronization of their activity (Gibson et al., 1999). In keeping with the diffuse connectivity into and within the inhibitory network, inhibitory interneurons project back onto excitatory principal neurons with exceptionally high probability, peaking between 50 and 100% connectivity with their immediately adjacent neighbors (Packer and Yuste, 2011; Levy and Reyes, 2012). Furthermore, these return projections do not discriminate between principal neuron ensembles (Fino and Yuste, 2011; Packer and Yuste, 2011), which suggests that the inhibitory network behaves as a powerful suppressive force in the cortical circuit; it is activated by any ensemble of principal neurons, and in turn inhibits all other ensembles.

Modeling studies have revealed that this recurrent network of excitatory and inhibitory interneurons is sufficient for generating gamma oscillations (Borgers et al., 2005; Oswald et al., 2009). *In vivo*, neocortical excitatory and inhibitory neurons fire at specific phases of the gamma oscillation (Hasenstaub et al., 2005). As the gamma oscillation approaches its negative trough (intracellular depolarization), principal neurons begin to fire, followed shortly after by inhibitory interneurons. In line with the sparse connectivity between principal neurons, they only fire sporadically during gamma oscillations, typically once every five to seven cycles. In contrast, inhibitory interneurons fire during most gamma cycles, which partly reflects their higher intrinsic excitability (McCormick et al., 1985) and innervation by principal neurons. This interplay can be initiated by punctate activation of principal neurons (Sohal et al., 2009). Indeed, Sohal and colleagues expressed channelrhodopsin (ChR2) in pyramidal neurons of the mouse prefrontal cortex, along with halorhodopsin (NpHR) in a subset of inhibitory interneurons. ChR2 activation of the pyramidal neurons elicited several cycles of gamma oscillations, which were suppressed by NpHR driven silencing of inhibitory interneurons.

The ubiquity of the network interactions described above suggests that gamma oscillations are fundamental to the telencephalon's operation. This has been borne out in numerous studies demonstrating gamma's mediation of synchrony both within and between cortical regions. For instance, Singer and colleagues demonstrated stimulus induced gamma synchronization for co-tuned populations of neurons within the primary visual cortex (Freiwald et al., 1995), across hemispheres (Engel et al., 1991a), and between primary and secondary visual cortices (Engel et al., 1991b). Underscoring the behavioral importance of gamma oscillations, attending to a stimulus enhances gamma synchrony both within (Fries et al., 2001) and between cortical regions (Gregoriou et al., 2009).

How would gamma oscillations promote coordination and integration of activity between regions? In particular, it is generally believed that the compression of ensemble activity into brief recurring epochs, in this case firing during a particular phase of gamma, facilitates postsynaptic depolarization through spatial integration (Konig et al., 1996; Volgushev et al., 1998; Salinas and Sejnowski, 2000). The temporal coordination through gamma provides a greater gain than independent increases in firing rate (Abeles, 1982; Kuhn et al., 2002).

Beyond their established role in coordinating inter-regional spiking, gamma oscillations are potentially important regulators of synaptic plasticity. However, the literature on this topic is sparse. Several forms of synaptic plasticity, such as associative long-term potentiation (Levy and Steward, 1983) and spike timing-dependent plasticity (Markram et al., 1997; Bi and Poo, 1998), depend on the coincidence of pre- and postsynaptic activity in a narrow temporal window, on the order of tens of milliseconds. Intriguingly, gamma oscillations regulate pre- and postsynaptic spiking on a similarly brief timescale, ~20 ms. In keeping with this, the direction of synaptic plasticity in cortical neurons is determined by the relative timing of excitatory postsynaptic potentials (EPSPs) to the phase of a 40 Hz postsynaptic subthreshold oscillation (Wespatat et al., 2004). In that study, potentiation occurred when EPSPs were coincident with the depolarizing peak of the oscillation, while coincidence with the hyperpolarizing peak lead to depression. An important caveat to these studies is that they rely upon highly stereotyped and repetitive stimulation protocols, which do not occur naturally in the nervous system. Thus, whether these experimental models engage the same plasticity mechanisms used *in vivo* remains unclear (Martinez and Derrick, 1996; Martin et al., 2000).

## Gamma oscillations and emotional memory

### Emotional stimuli and states

Returning to the topic at hand, if gamma oscillations are ubiquitous, why are they particularly relevant to emotional memory? There are several reasons immediately evident from the material reviewed thus far. First, EEG phenomena produced by arousing stimuli, such as cortical activation, likely reflect gamma oscillations. Second, gamma oscillations facilitate the coordination of signaling between brain regions, which is essential for the encoding and behavioral expression of memory. Lastly, attention, which figures prominently in several cognitive models of emotional behavior (e.g., Mather and Sutherland, 2011), enhances gamma oscillations and their coordinating abilities. These findings provide the basis for hypothesizing that the salience of emotional memory depends, in part, on the enhanced strength of gamma oscillations induced by emotional experience. Therefore, the rest of this review will explore the experimental evidence supporting this claim.

The ‘simplest’ forms of memory are those for individual stimuli (e.g., habituation, recognition), therefore gamma-band responses to single stimuli must be understood. After this, we will cover combinations of stimuli, where subjects learn that a neutral stimulus precedes an affective event. Each of the phases supporting this learning will be covered separately, namely acquisition, consolidation, and retrieval.

Stimuli are often dichotomized into two classes, neutral and affective. This is variously manifested across subfields; for instance, practitioners of associative learning divide stimuli into those that are *conditioned* and *unconditioned*. Unconditioned stimuli have affective qualities that typically drive behavioral responses without prior training. Depending on their intensity they can elicit emotional responses. In contrast, conditioned stimuli generally do not evoke a behavioral response, except when they are novel or out of place (Sokolov and Vinogradova, 1975).

Affective stimuli engender gamma oscillations in a variety of cortical and subcortical regions. In the human literature, numerous studies have demonstrated that the strength of gamma oscillations evoked by faces is partly determined by the emotions they express. For instance, Luo and colleagues were able to derive gamma-band activation in the amygdala from magnetoencephalographic (MEG) signals (Luo et al., 2007). During MEG recordings, subjects were required to identify the sex of faces that had a neutral, angry, or fearful expression. Fearful faces, in particular, produced enhanced gamma activation in the right amygdala within 30 ms of stimulus presentation. However, an important confound in studies of the neural correlates of image perception is that gamma oscillations have been linked to conscious awareness of visual stimuli (Tallon-Baudry and Bertrand, 1999; but see: Yuval-Greenberg et al., 2008). Thus, the gamma enhancement seen for fearful faces may arise from a potentially higher salience, independent of emotional valence. To rule this out, the conscious awareness of the stimuli and their emotional content have to be manipulated independently. If gamma-band activation reflects the emotionality of stimuli, then an effect of emotional content should be evident beyond the conscious awareness for the stimulus. To investigate this, Luo et al. (2009) presented subjects with neutral or fearful faces for either 30 (subliminal, unaware) or 100 ms (supraliminal, aware). Each face was preceded and followed by a 100 ms masking stimulus. Filler trials replaced the face with an empty oval. Subjects were asked to indicate whether a face had been present. Even for subliminal stimuli, there was a significant main effect of emotion in the gamma-band for visual, parietal, and posterior cingulate cortices, along with the right amygdala, spanning 30–140 ms post-stimulus onset. The enhancement of gamma by emotional faces has been confirmed repeatedly using MEG, scalp, and intracranial EEG (Balconi and Pozzoli, 2007; Jung et al., 2011; Sato et al., 2011; Senkowski et al., 2011; but see: Basar et al., 2008).

In addition to faces, other emotional pictorial stimuli can enhance gamma-band activity. Fortunately, many of the studies investigating this have drawn their stimuli from the International Affective Picture System (Lang et al., 1999), which facilitates comparisons between studies. For instance, Oya et al. (2002) studied patients with amygdala electrodes for presurgical mapping of intractable epilepsy. Patients passively viewed images that were either of positive, negative, or neutral emotional valence. LFP recording sites were selected for analysis only if they showed significant activation to any of the picture categories. Of these sites, only the negatively valenced stimuli elicited robust gamma activation in the amygdala. Besides the amygdala, cortical regions also exhibit enhanced gamma for pictures with affective qualities (Keil et al., 2001). In that study, subjects passively viewed pictures while EEG recordings were obtained. At posterior recording sites, negative affective stimuli produced an early onset (~80 ms) increase in the lower portion of the gamma-band. Starting at ~200 ms after stimulus onset, both negative and positive stimuli elicited increases in gamma-band power that lasted for several hundred milliseconds (see also: Müller et al., 1999; for a negative result see: Glauser and Scherer, 2008). Similar results were obtained with written words (Martini et al., 2012). Interestingly, gamma enhancement by emotional stimuli is reduced in patients with alexithymia, a condition characterized by a blunted ability to recognize and experience emotional states (Matsumoto et al., 2006). Overall, these results indicate that gamma-band activation in the amygdala and cortex tracks the emotionality of stimuli, particularly for unpleasant items, and independently of training.

Appetitive stimuli also elicit gamma oscillations. In 1960, Walter Freeman demonstrated that LFPs from the prepyriform cortex of awake cats exhibited enhanced gamma oscillations upon presentation of fish odor (Freeman, 1960). What makes this study compelling, especially given that stimulus salience is a potential confound, was that the cat's state affected the gamma response. If the same fish odor was presented to a satiated cat, then the gamma oscillations were severely attenuated, suggesting that the sensory aspects of the odor stimulus were not the principal drivers of gamma. Also arguing against a purely stimulus-driven interpretation of gamma is the finding that gamma oscillations in the amygdala of the cat are enhanced during periods of alertness or arousal (Pagano and Gault, 1964).

In addition, human gamma-band activation is related to overall anxiety level. For instance, Oathes et al. (2008) reported that entering an anxious state produced a sustained increase in gamma power. This effect was most prominent in the left temporal area, but precise localization was not possible due to the spatial limitations of EEG. Furthermore, subjects with generalized anxiety disorder, who experience disproportionate worrying, exhibit greater gamma power during worrying than in normal subjects. Finally, other studies have found tonic enhancements of gamma power during other emotional states. For instance, clinically depressed patients exhibit increased baseline levels of gamma-band power (Siegle et al., 2010). This finding is especially curious because depression has been associated with memory impairment (Burt et al., 1995). While a plethora of factors likely contribute to this deficit, increased gamma power may be one of them, which raises the question: how does this fit into the relationship between gamma oscillations and emotional memory? One explanation is that factors that enhance memory, such as level of arousal, can become impairing if they exceed a certain level, creating a so-called “inverted-U” relationship between their magnitude and behavioral performance (Yerkes and Dodson, 1908).

At this juncture it is worth noting that while this review focuses on gamma oscillations in the neocortex and amygdala (due to their well-known involvement in emotional memory), they have also been extensively investigated in the hippocampus (for reviews see: Colgin and Moser, 2010; Buzsaki and Wang, 2012), a structure implicated in learning and memory. While the importance of the hippocampus for emotion has been reinvigorated (e.g., Kimura, 1958; Fanselow and Dong, 2010), the relevance of gamma oscillations to this role has only been explored explicitly in a few studies. Lu et al. (2011) trained mice on a passive avoidance task, wherein mice delay entering a compartment in which they were previously shocked. Following training, the investigators measured LFPs from the CA3 region, and then obtained *ex vivo* hippocampal slices that exhibited spontaneous gamma oscillations. The degree of acquired avoidance to the shock compartment did not correlate with *in vivo* gamma strength, nor *ex vivo* spontaneous and kainate-induced gamma oscillations. A separate study measured the strength of gamma oscillations in *ex vivo* slices from mice that had undergone tone-shock classical conditioning (Albrecht et al., 2013). Fear conditioning tended to diminish the strength of kainate-induced gamma oscillations recorded from stratum radiatum and stratum lacunosum moleculare. These null findings do not offer a clear interpretation for the role of hippocampal gamma oscillations in emotional processing, especially given that transgenic mice with disrupted hippocampal gamma exhibit altered anxiety related behaviors (Fuchs et al., 2007; Dzirasa et al., 2011).

### Acquisition of emotional memory

Both in the laboratory and in nature, organisms must learn that certain neutral stimuli predict aversive or rewarding events. An especially well investigated form of such learning is classical conditioning, where an animal learns that a neutral cue (CS) predicts an aversive or rewarding event (US), leading to the development of a conditioned behavioral response. Acquisition is generally defined as the time period during which subjects develop a reliable conditioned response to the CS^1^.

Perhaps the first investigation focusing on gamma activation during the acquisition of classical conditioning was Miltner et al. (1999). For this study, human subjects were presented with two different colored lights, one of which terminated with an electric shock to the hand (CS+), while the other did not (CS−). Acquisition of the association was followed by an extinction phase. A series of questionnaires assessed the subject's preference for the CSs before training, after training, and after extinction. As expected, the subject's preference for the CS+ decreased following training, and this aversion diminished following extinction. EEG was recorded throughout the task and subjected to current source-density analysis (CSD) to improve the spatial resolution of the measured electrical activity. The authors sought to identify changes in CSD coherence between sites that tracked the acquisition of the contingency. Gamma power increased during presentation of either the CS+ or CS− at occipital recording sites, with a non-significant trend for greater gamma to the CS+. Gamma coherence was found to be enhanced to the CS+ between occipital, pericentral, and putative secondary somatosensory regions that were contralateral to the shocked hand. Enhanced coherence for the CS+ over the CS− was greatest near the end of stimulus, just prior to shock delivery. This temporal precision, in combination with the specificity for the CS+, and location of US delivery, suggests that this enhancement is not produced by a generalized arousal response, but instead reflects coordination between brain regions representing the CS+ and US. Moreover, coherence was strongest in the gamma frequency band, with neither the delta, theta, or alpha bands exhibiting a robust enhancement of coherence for the CS+ over the CS−. Extinction training reduced the enhanced coherence for the CS+, showing that the gamma coherence was not just determined by the physical properties of the CS+, but that it depended on the predictive relation between the CS+ and the shock.

A more recent study found that enhanced gamma power develops to a visual grating stimulus that is repeatedly paired with negative affective pictures (Keil et al., 2007). In this study, subjects were exposed to two grating stimuli oriented 90 degrees apart. These stimuli were presented without reinforcement during an initial baseline phase. On the following day, two training sessions occurred, wherein one of the gratings predicted the presentation of negative pictures, while the other did not. This was followed by an extinction session where the gratings were presented without reinforcement. Subjects acquired weak noise-induced startle responses to the grating that was paired with negative pictures, and this response was extinguished with extinction training. A majority of participants, however, could not verbally identify the grating that predicted negative pictures, and thus the modest startle response that was observed could have been driven by the subset of subjects that were aware of the contingency. Alternatively, the weak startle response may reflect unconscious processing of the cues. An unconscious mediation is possible given the evidence that circuits involved in threat processing, such as the amygdala, can operate for subliminal stimuli (see above). Gamma-band power was enhanced to the grating that predicted aversive pictures. Two gamma responses were produced, an early phase-locked evoked response, 60–90 ms after stimulus onset, and a longer latency induced response, that occurred 350–420 ms after stimulus onset. Importantly, only the evoked gamma-band response was enhanced by the grating predicting the aversive pictures. The induced response was evoked to both gratings, but despite its lack of stimulus specificity, it varied with training phase, peaking during initial acquisition, and then reducing across the second training session and extinction phase. This late response may best capture the mnemonic component of the task, because its lack of specificity comports with the lack of explicit memory for the task contingencies.

Overall, the studies reviewed above, and others (e.g., Jeschke et al., 2008), demonstrate that gamma oscillations occur during the acquisition of associations involving emotional stimuli. On the other hand, it is unclear if these same oscillations have an impact on subsequent memory. As reviewed below, a more successful paradigm for probing the impact of gamma oscillations on memory has been the subsequent memory effect (Paller and Wagner, 2002). In this analysis, stimuli or subjects are grouped by the strength of their memory following a training task. The neural signatures that were detected during training are then compared between groups, and a significant effect suggests that the neural signature in question has some relation with the encoding and storage of the memory. Gamma oscillations in the LFP and EEG exhibit subsequent memory effects for situations that lack emotional tone, a finding that has been demonstrated in humans with word (Fell et al., 2001; Sederberg et al., 2003, 2007) or pictorial stimuli (Osipova et al., 2006). Gamma-band synchrony between spiking activity and the LFPs in the macaque hippocampus also exhibits a subsequent memory effect for pictorial stimuli (Jutras et al., 2009). However, a drawback for many of these studies is that the time between encoding and retrieval was brief, at most an hour, and thus it is unclear if gamma at the time of encoding was important for long-term memory.

To overcome this, and to examine the relationship between gamma and emotional memory in particular, Headley and Weinberger (2011) recorded gamma oscillations from the auditory cortex of rats undergoing several daily sessions of tone/shock fear conditioning. In this task, a tone CS+ predicted the occurrence of a shock US, and the fear response to the tone was measured as a slowing of the heart rate during tone presentation, which reflects conditioned fear (Teyler, 1971). The auditory cortex is known to support fear memory for acoustic cues (Romanski and Ledoux, 1992; Boatman and Kim, 2006; Letzkus et al., 2011), beyond its obvious role in processing the tone CS+. During the first fear conditioning session, the degree of CS+ induced gamma-band activation to the tone had a positive relationship with the strength of conditioned responding on the subsequent day. After the first training day, gamma ceased to exhibit a subsequent memory effect, implying that gamma's relation to memory was confined to the acquisition phase of the task. Even so, gamma continued to occur during the remaining conditioning sessions, and so it might still have been able to facilitate new learning.

To test this, the same subjects underwent several sessions of discrimination conditioning; a novel tone was introduced that did not predict shock, the CS−. Subjects discriminated between the CSs; they maintained their fear responses to the CS+, and decreased responding to the CS−. However, not all subjects acquired this discrimination immediately. There was a range of performance, with some subjects still treating the CS− as if it predicted shock on the second discrimination training session. Curiously, these subjects exhibited stronger CS− induced gamma during the first discrimination training session. These results suggest that gamma serves as an *emotional tag*, perhaps in addition to its role in facilitating memory. Indeed, if gamma just supported memory for events as they happened irrespective of emotionality, then enhanced gamma to the CS− should have resulted in improved discrimination. Since this was not the case, the memory facilitation tied to gamma was related to the subjective emotional content of the experience.

This study also found that gamma-band activation modulated the magnitude of CS+ related plasticity in the auditory cortex. Previous work had established that fear conditioning with auditory stimuli shifts frequency tuning curves in auditory cortex so that the frequency of the CS+ elicits increased responses (Weinberger, 2004). This plasticity is evident soon after training, and persists for two months, the longest time point investigated so far (Weinberger et al., 1993), making it a candidate trace for emotional memory. In the Headley and Weinberger study (2011), changes in the tone frequency receptive fields were tracked across training days and correlated with CS+ induced gamma activation. It was found that gamma during the first training session predicted the subsequent change in the receptive field at the frequency of the CS+. Moreover, this predictive correlation was absent from all other conditioning sessions, suggesting that it was specific to the acquisition of the association. Taken together, these predictive correlations support the claim that gamma oscillations modulate the induction of long-term emotional memory.

Presumably the gamma oscillations generated in auditory cortex are facilitated by the activation of the cholinergic afferents projecting from the NB. Thus, with respect to the auditory cortex, direct NB stimulation should emulate an unconditioned stimulus (McLin et al., 2002). As reviewed above, NB stimulation produces gamma oscillations in the cortex, and NB receives direct projections from the amygdala. Rats that had been implanted with a stimulating electrode in NB and a recording electrode in auditory cortex underwent either paired or unpaired presentations of a tone and NB stimulation. After fifteen daily sessions of tone/NB pairing, subjects went through a generalization test, where tones of different frequencies, including the training tone, were presented without NB stimulation. The authors found that relative to tones of other frequencies, the tone that had been paired with NB stimulation gained a greater ability to evoke gamma-band activation. Furthermore, subjects that received unpaired presentations of the tone and NB stimulation did not develop enhanced gamma to the training tone, or any of the adjacent frequencies. Lastly, these results were mirrored in the behavior: presentation of the training tone elicited greater changes in heart rate and respiration than the untrained frequencies. However, it is unclear what the corresponding mnemonic content is for these responses. The behavioral responses observed to the training tone, which were an interruption in respiration and changes in heart rate, are the same as those seen with emotional learning. A follow up study that measured activation in other EEG frequency bands found that only gamma showed significant enhancement to the training tone in the paired group; all other bands either did not change or significantly decreased (McLin et al., 2003). Importantly, stimulation of the NB by itself produced gamma-band activation in auditory cortex, independently of overt movements or changes in behavioral state (Miasnikov et al., 2008). This suggests that the salient and arousing aspects of an emotional experience may be separable from the neural processes mediating its enhanced subsequent memory.

### Consolidation of emotional memory

Despite an abundant literature on the consolidation of emotional memories (McGaugh, 2000), no study has found a correlation between post-acquisition gamma oscillations and recall of emotional memories. The studies reviewed thus far focused on stimulus-driven gamma-band activation, which is easier to detect because one can average neural responses across trials, enhancing the signal to noise ratio. In contrast, the consolidation period lacks external events, demanding a more sophisticated analytical approach.

Overcoming this, Laitman et al. (2011) analyzed changes in gamma power surrounding the onset and offset of REM epochs during sleep in rats that underwent tone/shock fear conditioning. They found that fourteen days following fear conditioning, gamma power increased more rapidly during the transition to REM sleep, compared with the pretraining baseline. These changes were not present in subjects that received only shocks. Given the long interval between training and assessment, it is unclear whether this effect is related to memory consolidation. Another approach to study the role of gamma oscillations in memory consolidation is to probe the structures that have been tied to it. The amygdala's role in emotional memory consolidation is to orchestrate the activity of regions that presumably support long-term storage. This implies that amygdala activity is causally related to activity in presumptive storage structures during the consolidation period. Both the medial prefrontal cortex and hippocampus have been implicated in emotional memory. To investigate the amygdala's role in consolidation, Popa et al. (2010) trained rats on a fear conditioning task, and then recorded from the amygdala, hippocampus, and medial prefrontal cortex during post-training sleep. To assess interactions between these, they determined whether the change in freezing behavior, from the end of training to the recall test on the following day (i.e., consolidation) was correlated with coordination between the amygdala and these downstream regions during post-training sleep. They found that coordinated activity in the gamma-band between amygdala and hippocampus or amygdala and medial prefrontal cortex was not predictive, while activity in the theta band was, but only during REM sleep. These results stand as a robust, negative, finding for the involvement of gamma in emotional memory consolidation.

Complicating this picture is a study on gamma coherence during post-training slow wave sleep and memory for neutral word pair associations (Molle et al., 2004). Subjects were trained to associate word pairs, followed by a sleep period during which EEG recordings were obtained. A non-mnemonic form of the task was presented as well, where subjects attended to orthographic features of word stimuli. The memory group exhibited enhanced gamma coherence between EEG sites during post-training slow wave sleep. This was particularly strong between parietal/temporal and frontal sites. No such relationship was found for the subjects performing the non-mnemonic orthographic task.

Instead of correlating gamma oscillations during the consolidation period with subsequent memory, an alternative is to induce them in brain regions involved in consolidation of emotional memories. A recent study has done just that, using optogenetic tools to selectively excite neurons in the amygdala immediately after training rats on an inhibitory avoidance task (Huff et al., 2013). In inhibitory avoidance, the subject is placed in an alley divided in half, one side of which is darkened. When a rat is placed in the lighted half, his tendency will be to enter the dark compartment. Upon entering, the rat receives several foot shocks. During a subsequent test session, the rat is again placed in the lighted half and the latency to enter the dark compartment is measured. Conditioned fear manifests as an increased latency to enter the dark compartment. This task has been a mainstay for assessing emotional modulation of memory, in particular the role played by the amygdala. When administered shortly after acquisition of inhibitory avoidance, intraamygdalar injections of pharmacological agents that enhance or depress activity in the amygdala exert corresponding effects on subsequent memory (McGaugh, 2004).

Following up on these early findings, Huff et al. (2013) delivered different patterns of excitation to the amygdala immediately after training on inhibitory avoidance, with the goal of determining whether certain activity patterns are conducive to modulating the memory 24 h later. Of relevance to the role of gamma oscillations in emotional memory consolidation, they compared optogenetic stimulation of amygdala at either 20 or 40 Hz. They found significantly greater emotional memory for the 40 Hz stimulation subjects over the no stimulation condition, and a trend toward enhancement for 20 Hz stimulation. Complicating the interpretation of these findings, however, the 40 Hz group received more stimuli overall than the 20 Hz group, and thus it is unclear if the enhancement was a result of the frequency of the stimuli, and not just the number. Rectifying this would involve delivering the same number of stimuli, but at a different frequency, such as delivering the 20 Hz stimulation for twice as long as the 40 Hz. Despite this issue, the results still agree with the hypothesis that gamma oscillations modulate emotional memory consolidation.

The basolateral nucleus of the amygdala plays a critical role in the post-training consolidation of emotional memory (McGaugh, 2000). If such a role depends upon gamma oscillations, then the amygdala should be capable of generating them in the absence of training stimuli. This appears to be the case. Gamma oscillations in the amygdala occur spontaneously and exhibit coupling with neocortical activity, in the absence of overt stimuli (Collins et al., 2001), or even in the isolated slice with the application of agonists for glutamate (Randall et al., 2011) or cholinergic receptors (Sinfield and Collins, 2006). Moreover, the dependence of gamma oscillations in the amygdala on the cholinergic system (Popescu et al., 2009, reviewed below) may bridge them to the deep literature on hormonal regulation of amygdala function in consolidation. Indeed, blockade of cholinergic muscarinic receptors in the basolateral nucleus of the amygdala blocks the memory enhancing effects of glucocorticoids (Power et al., 2000).

### Expression of emotional memory

In contrast with the paucity of studies on the role of gamma on consolidation, their presence during the expression of emotional memories is well documented. An interesting observation to emerge from this work is that, just as affective stimuli enhance gamma oscillations, so do neutral stimuli that have come to signal affective events.

One of the first studies to show a relationship between gamma and performance on a task that could be construed as emotional was done in hungry dogs that acquired a cued instrumental response for food (Dumenko, 1995). It was found that once the dogs had acquired the contingency, the cue that indicated presence of the food evoked activation in the gamma-band.

However, perhaps more important than just demonstrating a correlation between gamma and emotional memory is to uncover its consequences for the circuits that support later performance and retrieval. Bauer et al. (2007) showed that the basolateral amygdala coordinates coherent gamma in the rhinal cortices. In this study, food-deprived cats were trained on a trace conditioning task with food reinforcement. Each trial was divided into three phases, a CS+ phase, indicating that food would soon be delivered, a delay period, and reward delivery. The gamma LFP coherence and spiking entrainment were assessed between the amygdala, perirhinal, and entorhinal cortices. Prior to conditioning, these regions exhibited LFP and unit gamma-band coherence. During initial conditioning, gamma coherence between regions did not change relative to baseline. However, by the late training sessions when performance had reached asymptote, LFP coherence between the three regions increased during the delay period and reward delivery. In parallel, unit activity in the rhinal cortices increasingly synchronized with amygdala gamma during the delay period, suggesting that the amygdala orchestrates cortical spiking on fast timescales during the performance of a well-learned appetitive task.

A similar pattern of results was also found when comparing activity in the amygdala and striatum (Popescu et al., 2009). Food restricted cats were trained on tone discrimination conditioning with food reinforcement, and over the course of several sessions learned that one of two tones predicted food delivery. Coherent gamma oscillations were present between the amygdala and striatum prior to training. Inactivation of the amygdala with the GABA agonist muscimol reduced gamma power in the striatum, implicating the amygdala as one of the sources of striatal gamma. Periods of high amplitude gamma in striatum also increased the strength of cross-correlations between striatal and amygdalar neurons. Importantly, training increased amygdalostriatal gamma-band coherence during the CS+, whereas reversing the CS contingencies decreased it.

Besides studies relying on appetitive training tasks, negative emotional learning has also been explored. Using four daily sessions of tone/shock classical fear conditioning, Headley and Weinberger (2013) tracked changes in the spectral content of LFPs during acquisition of conditioned fear, and the coordination of unit activity in primary auditory cortex. Recording sites were grouped based on the distance between preferred frequency and the CS+ frequency. Only gamma consistently increased with training, an effect seen irrespective of the recording sites preferred frequency. Yet, the enhancement tended to be stronger for sites with preferred frequencies close to the CS+. Where frequency tuning did matter was in the synchrony between unit activity and the phase of gamma oscillations (60 Hz band). Just as CS+ induced gamma power increased with training, so did the degree of phase-locking between unit activity and gamma. During the first training session, both the sites tuned near and away from the CS+ tended to decrease their phase-locking to gamma during CS+ presentation. By the last training session this had reversed, sites tuned near the CS+ now tended to increase their phase-locking with gamma during the CS+, while those tuned away had no net change. Does this change in phase-locking to gamma oscillations have consequences for the coordination of unit activity?

The synchrony arising from gamma oscillations is thought to support the binding of neurons into coactivated cell assemblies, allowing them to drive each other and their mutual downstream targets more effectively (Womelsdorf et al., 2007). Based on this work, modeling studies showed that both the phase and amplitude of gamma oscillations should modulate the correlation in spiking between neurons (Buehlmann and Deco, 2010). This was also found with the acquisition of conditioned fear. The strength of the cross correlation between unit activity at sites tuned near the CS+ frequency acquired enhanced modulation by both the phase and amplitude of CS+ induced gamma oscillations, while those tuned away from the CS+ frequency were not significantly affected.

## Conclusions

Bringing the reviewed studies together, several patterns emerge. The first is that affective stimuli, particularly those with negative valence, elicit enhanced gamma oscillations in both the neocortex and amygdala, the two structures that were the focus of this review. When these responses are *evoked*, having a short-latency and time-locked to stimulus onset, they exhibit modulation by emotional content that can be unconscious (Keil et al., 2007; Luo et al., 2009). Their short-latency suggests that this emotional modulation of gamma is bottom-up. *Induced* gamma on the other hand, which occurs several hundred milliseconds after stimulus onset and without phase-locking, reflects a subject's awareness of emotional content (Keil et al., 2007). In addition, induced gamma to training stimuli predicts memory one day later (Headley and Weinberger, 2011). The long latency of induced gamma is consonant with either top-down modulation or neuromodulatory influences.

Second, induced gamma is the form that appears most affected by learning. This is especially true for neutral events that have been paired with affective ones. The initially neutral stimuli acquire the ability to elicit stronger induced gamma oscillations. This increase typically occurs near the end of the stimulus, close to when the reinforcer would normally occur.

Third, the enhanced gamma that accompanies emotional learning appears to promote coordinated spiking between neurons. For example, this was the case for neurons in auditory cortex that responded to a fear conditioned stimulus (Headley and Weinberger, 2013), and those in peri- and entorhinal cortices with appetitive conditioning (Bauer et al., 2007). Gamma-driven increases in synchronized firing across a neuronal ensemble should allow representations of emotional stimuli to drive downstream targets more effectively (Fries, 2005).

Finally, there are presently four under-addressed issues in the study of gamma oscillations and emotional memory. The first is the dearth of studies investigating gamma's role in consolidation. Apart from the few reviewed above, to the best of our knowledge no other studies have probed this issue. The second issue is whether the function of gamma oscillations in emotional memory can be dissociated from their role in attention. Studies of gamma oscillations during emotional memory consolidation, when conducted during the post-training consolidation period, lack the discrete attention grabbing stimuli that are often reinforced. Thus, studies of gamma during this period may provide insights into their ability to coordinate activity without the ongoing influence of attentional processes. The third issue is the long-term consequences of the changes in gamma coordination that occur for stimuli that come to predict affective events. How does this enhanced gamma-band activation affect future learning about these stimuli, and are these changes in any way unique to emotional learning? Lastly, are gamma oscillations recruited in a qualitatively different manner during emotional experiences compared with non-emotional ones, or are these differences only quantitative? Perhaps emotional stimuli fall along a continuum that includes stimuli that are novel, goal-related, or devoid of emotional inflection, with each of these engaging circuits that mediate gamma oscillations to different degrees. Answering these questions could go a long way toward explaining the salience, vividness, and accessibility of emotional memories.

### Conflict of interest statement

The authors declare that the research was conducted in the absence of any commercial or financial relationships that could be construed as a potential conflict of interest.

## Footnotes

^1^Both empirically and conceptually it is difficult to define a dividing line between acquisition and expression. From a purely statistical standpoint, the statement that asymptotic performance has been reached is fundamentally probabilistic, simply stating that the chance that further enhancements reflect a continuing trend is highly unlikely, not that it is impossible. The division is also conceptually fraught, because expression obviously occurs during acquisition, since it is the increase in the responding, the expression, that defines that phase. A further complication is that subjects may acquire knowledge of the contingencies to be learned well before behavioral evidence is present for some response systems. For simplicities sake, the first training session will be treated as the acquisition phase, and the experimenter's chosen behavior will be assumed to adequately reflect memory.
